# Occupational performance characteristics in patients with attention‐deficit/hyperactivity disorder comorbid with major depressive disorders: Discriminant analysis with major depressive disorders

**DOI:** 10.1002/pcn5.70254

**Published:** 2025-11-20

**Authors:** Tomonari Hayasaka, Izumi Nagashima, Miku Hoshino, Koji Teruya, Yasuyuki Matumoto, Masami Murao, Taku Maruki, Masako Watanabe, Takeshi Katagiri, Yayoi Imamura, Mariko Kurihara, Yuki Oe, Yoshikazu Takaesu, Takashi Tsuboi, Koichiro Watanabe, Hitoshi Sakurai

**Affiliations:** ^1^ Department of Rehabilitation Kyorin University School of Health Sciences Tokyo Japan; ^2^ Department of Neuropsychiatry Kyorin University School of Medicine Tokyo Japan; ^3^ Kyorin University Hospital Tokyo Japan; ^4^ Kyorin University School of Health Sciences Tokyo Japan; ^5^ Department of Neuropsychiatry Graduate School of Medicine University of the Ryukyus Okinawa Japan

**Keywords:** artistic activity, attention‐deficit/hyperactivity disorder, discrimination analysis, major depressive disorder, occupational therapy

## Abstract

**Aim:**

Assessing symptoms and daily functioning in individuals with attention‐deficit/hyperactivity disorder (ADHD) comorbid with major depressive disorder (MDD) is challenging because of their limited self‐monitoring abilities. This study aimed to determine whether specific occupational behaviors could differentiate patients with ADHD comorbid with MDD from those with MDD alone.

**Methods:**

This study included patients who underwent a comprehensive assessment for suspected difficult‐to‐treat depression at Kyorin University Hospital, Japan. During an artistic activity program, three therapists evaluated occupational performance characteristics to identify potential traits. Characteristics in patients with comorbid ADHD and MDD were compared to those in MDD alone using the chi‐square test. Key discriminators were selected based on significant characteristics (*p* < 0.01). Discriminant analysis was conducted to enhance group differentiation, with prediction accuracy assessed via area under the curve (AUC). This study was approved by the Research Ethics Committee.

**Results:**

A total of 71 occupational performance characteristics were identified through retrospective analysis, of which 12 showed significant differences between 29 patients with comorbid ADHD and MDD and 35 patients with MDD alone. Four variables were selected as independent predictors in the discriminant analysis: “Have its own manners,” “Feel uncomfortable in groups,” “Work only at your own pace,” and “Ask questions and consult.” These variables contributed to the construction of a linear discriminant function. The discriminant model yielded a Wilks' *λ* of 0.631 (*p* < 0.001) and achieved a classification accuracy of 81.3%. The receiver operating characteristic (ROC) curve showed an AUC of 0.857, with a sensitivity of 72.4% and a specificity of 88.6%.

**Conclusion:**

These findings highlight the role of occupational performance characteristics in differentiating comorbid ADHD and MDD from MDD alone. This approach may contribute to more tailored treatment strategies.

## Graphical abstract

This retrospective study identified four occupational performance characteristics that distinguished individuals with ADHD and comorbid MDD from those with MDD alone, achieving 81.3% accuracy (area under the curve [AUC] = 0.857). These findings support the potential of incorporating occupational profiles into assessments to improve diagnostic precision and guide individualized, function‐based interventions.

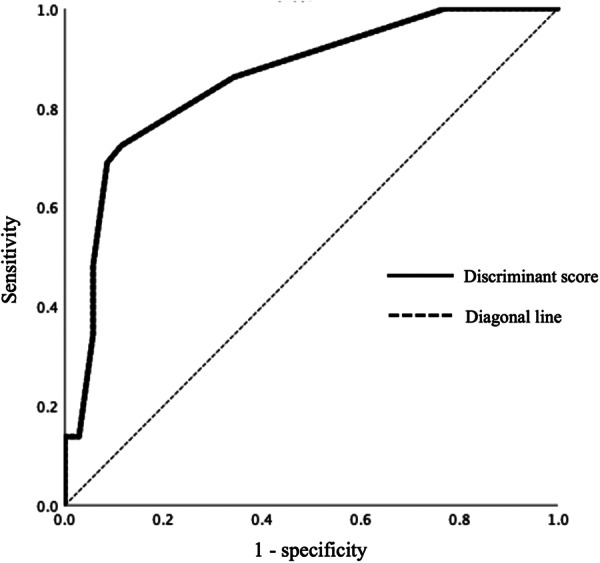

## INTRODUCTION

Attention‐deficit/hyperactivity disorder (ADHD) is a prevalent neurodevelopmental disorder, affecting approximately 7.2% of children and 2.5% of adults worldwide.^1^ Characterized by persistent symptoms of inattention, hyperactivity, and impulsivity, ADHD often leads to substantial functional impairments across multiple domains of daily life.^2^, ^3^, ^4^ These impairments can interfere with academic achievement, occupational performance, and social relationships, frequently resulting in chronic stress, diminished self‐esteem, and overall reduced quality of life.^5^, ^6^ Importantly, individuals with ADHD are at increased risk for developing major depressive disorder (MDD), with numerous studies reporting high rates of comorbidity between the two conditions.^7^, ^8^ The coexistence of ADHD and MDD is associated with more severe symptomatology, poorer treatment response, and greater functional impairment compared to either disorder alone.^9^, ^10^, ^11^ This comorbidity amplifies the overall burden of illness, contributing to worse psychosocial outcomes and presenting considerable challenges to public health and healthcare systems.^12^, ^13^, ^14^

When assessing comorbidity in patients with suspected ADHD, the Adult ADHD Self‐Report Scale (ASRS) and the ADHD Rating Scale are commonly used.^15^, ^16^ Although the reliability of self‐reported data may be compromised, individuals with ADHD, particularly those with co‐occurring depression, struggle to accurately perceive and report their own behaviors.^17^ The reliability of reports from parents and other informants reflects behavioral characteristics observed primarily within the limited context of the home environment, which may result in incomplete or restricted information.^18^, ^19^ Given these limitations, objective assessments of attention and behavior conducted by healthcare professionals in settings may provide more accurate insights into the manifestations of ADHD.^20^, ^21^

Occupational therapy is a case‐centered intervention aimed at enhancing participation in meaningful daily activities and improving overall functional capacity.^22^ In individuals with ADHD, the primary goals of occupational therapy include promoting adaptive skills, supporting engagement in daily routines, and improving executive functioning through structured and purposeful activities.^23^, ^24^ This therapeutic approach enables practitioners to evaluate how ADHD symptoms manifest across real‐life contexts, such as home, school, or workplace environments, thus providing a comprehensive understanding of functional impairments.^25^ Accordingly, prior studies have investigated the effectiveness of occupational therapy in managing core ADHD symptoms and in improving social and academic functioning.^26^, ^27^ Nevertheless, to our knowledge, no study has specifically examined occupational performance characteristics that differentiate individuals with comorbid ADHD and MDD from those with MDD alone. Given the high prevalence and clinical significance of this comorbidity, along with the existing gaps in current knowledge, identifying distinct patterns of occupational functioning may provide critical insights for both assessment and intervention strategies.

To address this gap, we conducted a retrospective study to investigate whether specific occupational performance profiles can distinguish individuals with comorbid ADHD and MDD from those diagnosed with MDD alone.

## METHODS

### Study design

This retrospective study analyzed data from patients who underwent a comprehensive assessment for difficult‐to‐treat depression at Kyorin University Hospital in Tokyo, Japan, between January 2015 and March 2019. In the present study, “difficult‐to‐treat depression” was defined as depression in patients who continued to experience persistent symptoms and failed to achieve full remission despite having received standard treatment at other institutions. The study description was detailed elsewhere.^28^ Briefly, the hospitalization for this examination followed a 1‐week program. Patients underwent a series of mental function tests, the results of which were reviewed by a multidisciplinary team for diagnosis and treatment planning. These findings were then communicated to the patients. The program also included a behavioral assessment conducted through occupational therapy. We conducted a retrospective analysis of medical records to explore how occupational therapy documentation could contribute to a deeper understanding of ADHD with comorbid MDD. All patients with difficult‐to‐treat depression were given the option to opt out. The study was approved by the Faculty of Medicine Research Ethics Committee, Kyorin University (approval number: R03‐096).

### Participants

For the participant, the inclusion criteria were as follows: (a) Patients who underwent a detailed assessment for difficult‐to‐treat depression during the 1‐week diagnostic hospitalization, (b) an age range of 20–70 years, (c) a diagnosis of MDD, based on the Diagnostic and Statistical Manual of Mental Disorders, Fifth Edition (DSM‐5), and (d) a MADRS (Montgomery‐Åsberg Depression Rating Scale) score of ≥7. The diagnosis of ADHD was made according to the DSM‐5 diagnostic criteria during the 1‐week diagnostic hospitalization. As a supplementary diagnostic measure, ASRS was administered. These assessments were conducted by psychiatrists and clinical psychologists. Exclusion criteria for both groups included: (a) ongoing alcohol or substance abuse, (b) an imminent risk of suicide, and (c) a severe physical illness.

### Occupational therapy program description

Occupational performance characteristics were evaluated during a single session of an artistic activity program in occupational therapy, with each participant attending only once. Before the session, participants were individually interviewed and informed that the activity was intended solely for behavioral assessment, not for therapeutic purposes. To ensure consistency and minimize variability between the two groups, session procedures including the introduction, facilitation, and recording were standardized (Table 1). The program offered a variety of creative activities, such as coloring, origami, knitting, and leatherwork, allowing participants to choose from over ten tasks of varying difficulty levels. Additionally, participants documented their self‐evaluation, mood status, and pre‐ and post‐session impressions using a scale from 1 (very poor) to 5 (excellent).

**Table 1:** Implementation of artistic activity program.

Procedure	Details
(a) Start of the program	– Promotion of motivation for participation – Confirmation of participation
(b) Description of the program	– Confirmation of content and purpose of the session
(c) Icebreaker	– Self‐introduction of each participant
(d) Selection of artwork task	– Free selection of tasks from more than 10 choices with different levels
(e) Implementation of the artwork task	– Preparation of tools and materials – Creation of the selected
(f) End of the program	– Present impressions for the session – Cleaning up

*Note*: The program was conducted in group sessions of approximately 10 participants.

### Occupational performance characteristics evaluation

Occupational therapists observed participants' communication skills, task engagement, and ability to adapt to the environment. After each session, they evaluated and documented participants' occupational performance characteristics. The behavioral assessments in occupational therapy were independently recorded by each occupational therapist. These records were then analyzed and categorized using a content analysis approach by three occupational therapists. The classification process followed five steps and was conducted collaboratively. This process involved (1) extracting occupational performance characteristic‐related information from records, (2) removing unnecessary content to create raw data, (3) substantially condensing this data, (4) organizing it based on semantic similarities and converting it into equivalent but unaltered expressions, and (5) categorizing these into occupational performance characteristics. The occupational performance characteristics score was assigned using a binary classification method based on its presence or absence. In cases of disagreement, the therapists reviewed differing perspectives and reached a consensus on the most appropriate classification.

### Statistical analysis

Baseline demographic and clinical data, including age, gender, and educational background, were extracted from medical and program records related to the artistic activity. Additional data collected for each participant included the duration of illness, the MADRS score, the YMRS (Young Mania Rating Scale) score, the ASRS score, the AQ (Autism‐Spectrum Quotient) score, and relevant occupational performance characteristics. To compare the ADHD with comorbid MDD (ADHD‐MDD) patient group to the MDD patient group across these variables, the Mann–Whitney *U* test was used for continuous variables, while categorical variables were analyzed using either the *χ*² test or Fisher's exact test, as appropriate. To further differentiate between the two groups, discriminant analysis was performed using the stepwise method. Independent variables included occupational performance characteristics that were statistically significant (*p* < 0.01) in the chi‐square test. Receiver operating characteristic (ROC) curves were generated to assess the accuracy of these classifications. Predictive performance was measured using the area under the curve (AUC), with classifications as follows: high (AUC > 0.9), moderate (0.7 ≤ AUC < 0.9), and low (0.5 ≤ AUC < 0.7).^29^ All statistical analyses were conducted using spss version 28 for Windows (spss Inc., NY), with a *p*‐value of less than 0.05 considered statistically significant.

## RESULTS

A total of 29 ADHD‐MDD patients (14 males, average age = 40.3 ± 12.7 years) and 35 MDD patients (14 males, average age = 41.1 ± 12.7 years) met the criteria. Baseline demographic and clinical variable comparisons between the ADHD‐MDD patient group and the MDD patient group revealed significant differences in the YMRS scores (*p* < 0.01) and applicable occupational performance characteristic (*p* < 0.001), with the ADHD‐MDD patient group demonstrating higher rates in both categories (Table 2). No significant differences were found in other variables.

**Table 2:** Comparison of demographic and clinical valuables between the attention‐deficit/hyperactivity disorder with comorbid major depressive disorder (ADHD‐MDD) patient group and major depressive disorder (MDD) patient group.

Variables	Mean ± SD or *n* (%)		*p*‐Value
	ADHD‐MDD patient group (*n* = 29)	MDD patient group (*n* = 35)	
Age (years)	40.3 ± 12.7	41.1 ± 12.7	0.845
Male	14 (48.3%)	14 (40.0%)	0.506
University degree	14 (48.3%)	18 (51.4%)	0.910
Disease duration (years)	12.2 ± 7.2	9.7 ± 8.0	0.111
MADRS scores	21.5 ± 8.1	21.2 ± 8.0	0.995
YMRS scores	2.7 ± 2.5	1.4 ± 2.6	0.005**
ASRS scores	3.5 ± 1.7	2.8 ± 1.8	0.089
AQ scores	23.0 ± 8.4	22.8 ± 6.7	0.967
Number of applicable occupational performance characteristics	13.3 ± 3.5	10.8 ± 3.7	0.007**

Abbreviations: AQ, Autism‐Spectrum Quotient; ASRS, Adult ADHD Self‐Report Scale; MADRS, Montgomery Åsberg Depression Rating Scale; YMRS, Young Mania Rating Scale.

** *p* < 0.01.

A total of 71 occupational performance characteristics were identified across both groups (Table S1). Of these, 12 characteristics exhibited significant differences between the groups (Table 3). Individuals in the ADHD‐MDD patient group were more likely to “Have their own manners,” “Ask questions and seek consultation,” and “Work only at their own pace,” whereas individuals in the MDD patient group were more likely to “Feel uncomfortable in groups.”

**Table 3:** Comparison of occupational performance characteristics between the attention‐deficit/hyperactivity disorder with comorbid major depressive disorder (ADHD‐MDD) patient group and major depressive disorder (MDD) patient group.

Variables	Participants in each occupational performance characteristic, *n* (%)		*p*‐Value
	ADHD‐MDD patient group (*n* = 29)	MDD patient group (*n* = 35)	
Have its own manners	16 (55.2%)	6 (17.1%)	0.002**
Feel uncomfortable in groups	0 (0.00%)	8 (22.9%)	0.005**
Ask questions and consult	18 (62.1%)	10 (28.6%)	0.007**
Work only at your own pace	9 (31.0%)	2 (5.7%)	0.009**
Work on your own without using a textbook	7 (24.1%)	1 (2.9%)	0.013*
Difficulty in handling some tools	5 (17.2%)	0 (0.0%)	0.016*
Feel down and decrease self‐esteem	9 (27.6%)	3 (0.0%)	0.024*
Have negative remarks (e.g., “I can't.”)	2 (6.9%)	10 (28.6%)	0.027*
Concentrate on work	17 (58.6%)	11 (31.4%)	0.027*
Obvious fatigue	12 (41.4%)	6 (17.1%)	0.031*
Messy (i.e., difficulty in organizing things)	4 (11.4%)	0 (0.0%)	0.037*
High self‐esteem	7 (24.1%)	2 (5.7%)	0.040*

* *p* < 0.05,

** *p* < 0.01.

**Figure 1:**
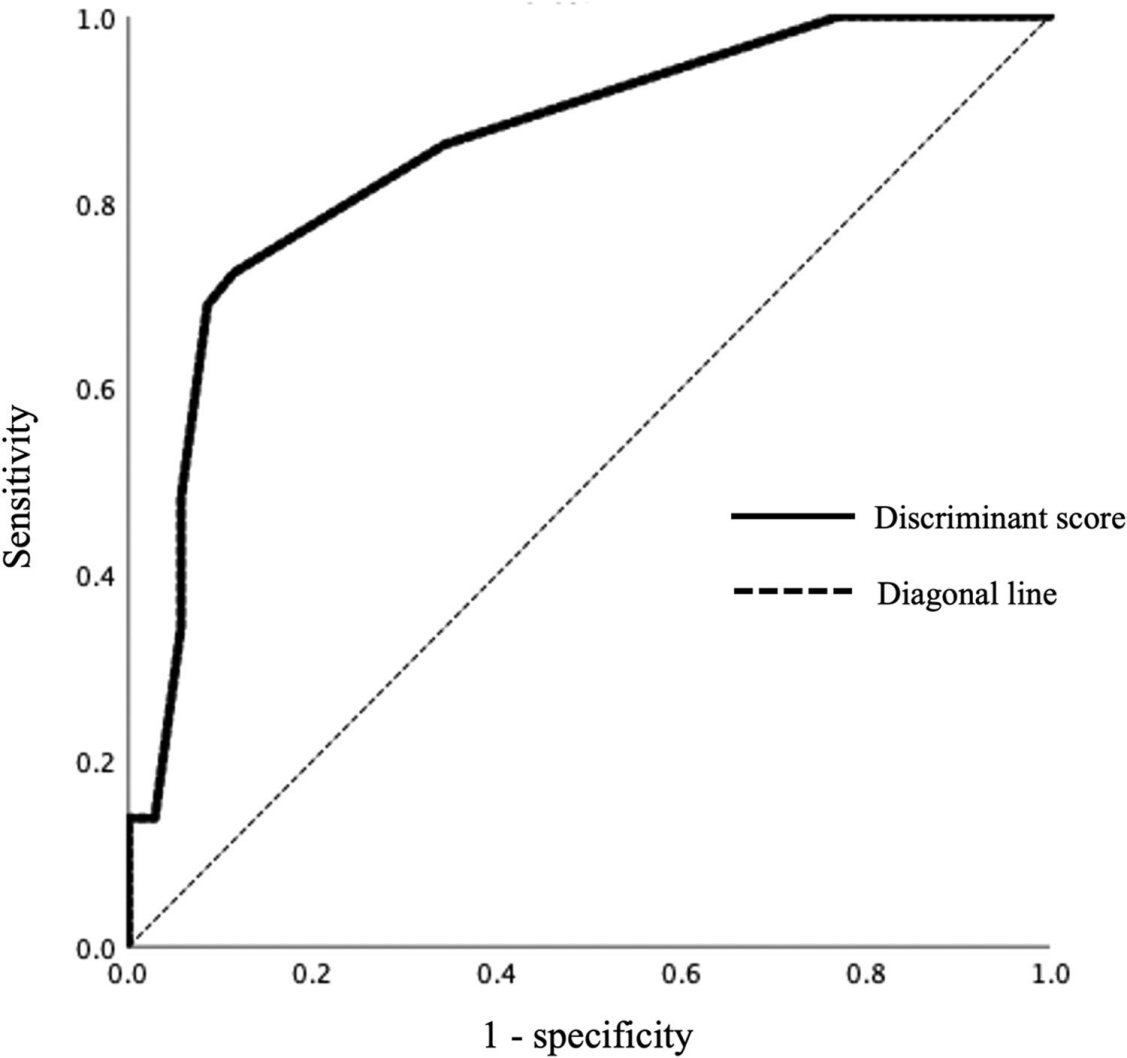
Receiver operating characteristic curves of discriminant score.

Discriminant analysis was conducted using the distinction between the ADHD‐MDD patient group and the MDD patient group as the dependent variable, and the aforementioned four variables as independent variables. To ensure the ROC curve was consistently oriented, the score for “Feel uncomfortable in groups” was reversed, giving all data positive discriminant scores. The results indicated that these variables produced a linear discriminant formula: 11.578 × “Have its own manners” + 1.696 × “Feel uncomfortable in groups (*reversal item)” + 1.074 × “Work only at your own pace” + 0.757 × “Ask questions and consult” − 2.543. Wilks' *λ* was 0.631 (*p* < 0.001), and the true discrimination rate was 81.3% (Table 4). When the ROC curve for discriminating the MDD patient group from the ADHD with comorbid MDD patient group was constructed using the discriminant scores obtained from the linear discriminant formula, the AUC was 0.857 (95% CI = 0.762–0.951, *p* < 0.001), indicating very high accuracy, with sensitivity and specificity of 72.4% and 88.6%, respectively, at a cutoff value of 0.479 (Figure 1).

**Table 4:** Discriminant analysis by occupational performance characteristics.

Analytic factors	Standardized canonical discriminant function coefficient	Canonical discriminant function coefficient	*p*‐Value
Have its own manners	0.698	1.578	0.001**
Feel uncomfortable in groups	0.535	1.696	0.005**
Work only at your own pace	0.388	1.074	0.007**
Ask questions and consult	0.360	0.757	0.007**

*Note*: These variables produced a linear discriminant formula: 1.578 × “Have its own manners” + 1.696 × “Feel uncomfortable in groups (*reversal item)” + 1.074 × “Work only at your own pace” + 0.757 × “Ask questions and consult” − 2.543. The linear discriminant function was developed by calculating discriminant scores from the data obtained using the binary classification method of the occupational performance characteristic. The score for “Feel uncomfortable in groups” was converted from negative to positive values to ensure consistency in the reporting of the receiver operating characteristic (ROC) curve.

** *p* < 0.01.

## DISCUSSION

To the best of our knowledge, this is the first retrospective study to investigate behavioral characteristics that distinguish patients with comorbid ADHD and MDD from those with MDD alone. Discriminant analysis identified four occupational performance characteristics that significantly differentiated the two groups: “Have its own manners,” “Feel uncomfortable in groups (*reversed item),” “Work only at own pace,” and “Ask questions and consults.” These variables were used to construct a linear discriminant function: 1.578 × “Have its own manners” + 1.696 × “Feel uncomfortable in groups (*reversed item)” + 1.074 × “Work only at own pace” + 0.757 × “Ask questions and consult” – 2.543, resulting in a discrimination accuracy of 81.3%. These findings have important implications for clinical practice, suggesting that specific patterns of occupational behavior may aid in the diagnostic differentiation of comorbid ADHD and MDD, and inform the tailoring of individualized intervention strategies.

The four occupational behavior traits identified in the present study are related to ADHD characteristics and provide important insight into the meaning of ADHD comorbidity in depression. The characteristic “Have its own manners” likely captures the distinctive mannerisms and behavioral tendencies often observed in individuals with ADHD, which stem from impairments in executive functioning and self‐regulation.^2^, ^30^ Difficulties with attention, planning, and cognitive flexibility may lead these individuals to develop personal routines or ritualized behaviors as compensatory strategies, particularly when adhering to external instructions is challenging.^31^ While such behaviors may appear maladaptive, they often serve to support task engagement and preserve self‐efficacy under conditions of cognitive strain. In the presence of comorbid depression, low self‐esteem may be further exacerbated, intensifying the drive to avoid failure and reinforcing rigid adherence to individualized behavioral patterns as a means of maintaining emotional stability.^32^ It is also noteworthy that these behavioral characteristics are unlikely to emerge during brief interviews or activities and are often revealed as individuals become acclimated to the setting, particularly during prolonged engagement.

The reversed item “Feel uncomfortable in groups” may reflect cognitive characteristics commonly associated with ADHD, such as impairment in self‐monitoring and difficulties in sustaining attention.^33^ These traits can interfere with the development of social connectedness in group contexts and may reduce individuals' awareness of their own subjective experiences, including feelings of discomfort or alienation in social situations. Individuals with ADHD often have difficulty regulating interpersonal boundaries and directing attention toward social cues, which can impair their sense of belonging and their ability to recognize social discomfort.^34^ Additionally, deficits in attentional control may compromise both self‐monitoring and responsiveness to social feedback, contributing to a diminished capacity for self‐awareness.^35^ Therefore, behavioral changes necessary for social adaptation are often difficult to achieve, partly because individuals themselves are frequently unaware of the need for such changes.

The characteristic “Work only at own pace” is likely rooted in impairments in executive functioning and the emergence of avoidant behavioral patterns.^30^, ^36^ Core executive functions, such as sustained attention, planning, and cognitive flexibility, are often compromised in individuals with ADHD. When depressive symptoms are present, reductions in motivation and concentration may further exacerbate difficulties in maintaining pace with others,^37^ leading to a strong reliance on a self‐directed rhythm during task execution.^38^ Moreover, individuals with ADHD frequently experience repeated failures from an early age, which can contribute to chronic low self‐esteem. As a result, they may adopt avoidant strategies,^39^, ^40^ such as rigidly adhering to their own approach to tasks, as a means of coping. This behavior may represent an adaptive response to neurocognitive vulnerabilities, further reinforced by accumulated negative experiences and characterized by a tendency to avoid risk and failure.

The item “Ask questions and consult” indicates that patients with comorbid ADHD and MDD are more likely than those with MDD alone to seek assistance or clarification when they do not understand a task. This behavior likely arises from fundamental characteristics of ADHD and may reflect a coping strategy rooted in impulsivity.^41^ The structured and supportive environment of the hospital setting, where patients could expect a response when reaching out, may have further facilitated this impulsive help‐seeking behavior. Additionally, a cross‐sectional survey of the adult population has shown that individuals with comorbid ADHD and depression tend to score higher on the YMRS score compared to those with depression alone.^42^ A similar trend was observed in the present study, suggesting that impulsivity related to ADHD may be misinterpreted or self‐reported as hypomanic symptoms. As a result, a significant difference may have existed in the number of applicable occupational performance characteristics. Conversely, this occupational performance characteristic may also be interpreted as a strength, particularly in the context of help‐seeking behavior, and could reflect a specific stress‐coping strategy.^43^

In this study, no significant difference was observed in the total ASRS scores between the two groups. This may be because the ASRS is a self‐report screening tool, and its accuracy depends on patients' self‐awareness: therefore, it may fail to capture symptoms in individuals with limited insight. Patients with ADHD often underestimate their attentional and hyperactive‐impulsive symptoms, many of which are more apparent to others than to themselves.^33^, ^37^ Moreover, depressive symptoms such as concentration problems and restlessness overlap with ADHD features, while negatively biased self‐assessment in depression may elevate ASRS scores in non‐ADHD patients, thereby reducing between‐group differences.^35^ Nevertheless, the AUC of the ROC curve based on the discriminant score was 0.857, indicating a moderate level of diagnostic accuracy. The ability to distinguish between patients with comorbid ADHD and MDD and those with MDD alone based on occupational performance characteristics carries important clinical implications. Incorporating these features into standardized assessment tools could enhance diagnostic accuracy and support the formulation of more personalized, functionally oriented treatment plans. Recognizing specific behavioral patterns, including difficulties in social interaction, task persistence, and self‐regulation, can help clinicians more effectively identify areas of functional impairment that may otherwise be overlooked in conventional psychiatric interviews. Occupational therapy provides a valuable and ecologically valid setting for evaluating these behaviors, as it allows observation of real‐time actions in a structured yet naturalistic context.^22^, ^24^ These insights may inform more individualized and effective treatment strategies, tailored to each patient's specific needs.

Despite the promising findings, this study has several limitations that should be addressed in future research. First, the sample size was relatively small, and the study was conducted at a single institution, which limits the generalizability of the results. A multicenter study with a larger sample would enhance the external validity of these findings. Second, although the occupational performance characteristics were identified through retrospective analysis, future research should examine these characteristics in more naturalistic settings to assess their predictive validity for real‐world functioning. Third, the selection of these characteristics was based on a retrospective survey, which may have introduced bias in both classification and data quality. Fourth, the study relied on assessments conducted within the context of an occupational therapy program, which may not fully reflect the breadth of individuals' daily functioning. Moreover, because these assessments were developed at the discretion of individual therapists, their standardization and generalizability may be limited. Fifth, in this study, the YMRS score and the number of applicable occupational performance characteristics were considered potential confounding factors that may also impact behavioral characteristics. Sixth, although the use of psychostimulant or non‐stimulant ADHD medications was not confirmed in the MDD patient group, one participant in the ADHD‐MDD patient group was receiving these medications. The potential effects of psychostimulant and non‐stimulant ADHD medications on occupational performance could not be determined within the scope of the present study. Finally, the relationships between the various occupational performance characteristics could not be fully examined.

In conclusion, this study provides valuable insights into how specific occupational performance characteristics can be used to differentiate individuals with comorbid ADHD and MDD from those with MDD alone. Specific occupational performance characteristics were found to be significant for differentiation and could be utilized in clinical settings to improve diagnostic accuracy and enhance treatment strategies. Future research should explore the application of these findings in broader clinical populations, including those with other comorbid mental health conditions, to further understand the utility of occupational performance characteristics in mental health assessment and intervention.

## AUTHOR CONTRIBUTIONS

**Tomonari Hayasaka**: Acquisition and analysis of data; drafting the manuscript. **Izumi Nagashima**: Acquisition and analysis of data; revising the manuscript. **Miku Hoshino**: Acquisition and analysis of data. **Koji Teruya**: Acquisition and analysis of data. **Yasuyuki Matumoto**: Acquisition and analysis of data. **Masami Murao**: Acquisition and analysis of data. **Taku Maruki**: Acquisition and analysis of data. **Masako Watanabe**: Acquisition and analysis of data. **Takeshi Katagiri**: Acquisition and analysis of data. **Yayoi Imamura**: Acquisition and analysis of data. **Mariko Kurihara**: Acquisition and analysis of data. **Yuki Oe**: Acquisition and analysis of data. **Yoshikazu Takaesu**: Acquisition and analysis of data. **Takashi Tsuboi**: Acquisition and analysis of data. **Koichiro Watanabe**: Acquisition and analysis of data. **Hitoshi Sakurai**: Conception and design of the study; acquisition and analysis of data.

## CONFLICT OF INTERESTS STATEMENT

Dr. Tomonari Hayasaka, Dr. Izumi Nagashima, Ms. Miku Hoshino, Dr. Koji Teruya, Dr. Taku Maruki, Dr. Masako Watanabe, Dr. Takeshi Katagiri, Dr. Yayoi Imamura, Ms. Mariko Kurihara, and Mr. Yuki Oe have nothing to declare. Dr. Masami Murao received honorariums from Sumitomo Pharma and Yoshitomiyakuhin. Dr. Yasuyuki Matumoto received grants from the Japan Society for the Promotion of Science and honorariums from Sumitomo Pharma, Janssen Pharmaceutical, and Meiji Seika Pharma. Dr. Takaesu has received lecture fees from Takeda Pharmaceutical, Otsuka Pharmaceutical, Daiichi Sankyo Company, Shionogi, Mochida Pharmaceutical, Lundbeck Japan, Eisai, MSD, and Viatris Pharmaceuticals, outside the submitted work, and research funding from Otsuka Pharmaceutical, Meiji Seika Pharma, MSD, and Eisai. Dr. Takashi Tsuboi received grants from the Japan Society for the Promotion of Science and honorariums from Takeda Pharmaceutical, Otsuka Pharmaceutical, Meiji Seika Pharma, Shionogi Pharma, Yoshitomiyakuhin, Sumitomo Pharma, Kyowa Pharmaceutical, MSD, Nippon Boehringer lngelheim, Mylan EPD, Mitsubishi Tanabe Pharma, Viatris, Mochida Pharmaceutical, Janssen Pharmaceutical, TEIJIN PHARMA, and Lundbeck Japan. Dr. Koichiro Watanabe is a consultant of Boehringer Ingelheim, Daiichi Sankyo, Eisai, Eli Lily, Janssen Pharmaceutical, Kyowa Pharmaceutical, Lundbeck Japan, Luye Pharma, Mitsubishi Tanabe Pharma, Otsuka Pharmaceutical, Lundbeck Japan, Luye Pharma, Mitsubishi Tanabe Pharma, Otsuka Pharmaceutical, Pfizer, Sumitomo Dainippon Pharma, Taisho Pharmaceutical, and Takeda Pharmaceutical. Dr. Hitoshi Sakurai received grants from the Japan Society for the Promotion of Science, Japan Research Foundation Clinical Pharmacology, and Takeda Science Foundation, and honorariums from Eisai, Takeda Pharmaceutical, Otsuka Pharmaceutical, Meiji Seika Pharma, Shionogi Pharma, Yoshitomiyakuhin, Sumitomo Pharma, Kyowa Pharmaceutical, MSD, Viatris, Mochida Pharmaceutical, and Lundbeck Japan.

## ETHICS APPROVAL STATEMENT

This study was approved by the institutional review board of the School of Medicine, Kyorin University (R03‐225).

## PATIENT CONSENT STATEMENT

Participants diagnosed were given the option to opt out.

## CLINICAL TRIAL REGISTRATION

N/A.

## Supporting information

Supporting Information.

## Data Availability

The data that support the findings of this study are openly available in figshare at https://doi.org/10.6084/m9.figshare.29436512.v1.
