# Supplementary material for: Occupational performance characteristics in patients with attention‐deficit/hyperactivity disorder comorbid with major depressive disorders: Discriminant analysis with major depressive disorders
Source: PCN Rep. 2025 Nov 20;4(4):e70254. doi: 10.1002/pcn5.70254 (PMC12631064; doi:10.1002/pcn5.70254)
Supplement: Supplementary file 1 — Supporting Information. [file PCN5-4-e70254-s001.docx]

Supplementary Table 1. Occupational performance characteristics evaluated through an artistic activity program in occupational therapy

| **No.** | **OPC (No.1-18)** | **No.** | **OPC (No.19-36)** | **No.** | **OPC (No.37-54)** | **No.** | **OPC (No.55-69)** |
| --- | --- | --- | --- | --- | --- | --- | --- |
| 1 | Accept suggestions from others | 19 | Difficulty in understanding procedures | 37 | Lots of comments in OT note | 55 | Self-rating 2 levels up after OT |
| 2 | Altruism | 20 | Discard the work without any attachment | 38 | Makeshift work | 56 | Shy during conversations |
| 3 | Ask questions and consult | 21 | Dislike (or state dislike for) artistic activities | 39 | Messy (i.e. difficulty in organizing things) | 57 | Soliloquize while working |
| 4 | Bring personal items into the OT room | 22 | Excessive confirmation | 40 | Messy work | 58 | Start to talk to others once adapted |
| 5 | Calm as you get used to the place | 23 | Excessive reactions to others | 41 | Misunderstand processes | 59 | State that OT is fun |
| 6 | Careful and attentive in working | 24 | Expressionless | 42 | Nervous in interpersonal interactions | 60 | Sudden interruption of work |
| 7 | Change activities frequently | 25 | Feel down and decrease self-esteem | 43 | Never or rarely converse with others | 61 | Superficial communication |
| 8 | Choose or seek new activities | 26 | Feel uncomfortable in groups | 44 | No eye contact | 62 | Take a break during the program |
| 9 | Choose simple work | 27 | Fluent conversations | 45 | Not seek help | 63 | Take a long time to be seated |
| 10 | Clean up or help clean up | 28 | Frequent changes in emotions during activities | 46 | Obvious fatigue | 64 | Try to get others to do their work for them |
| 11 | Clearly uplifting mood | 29 | Give instructions to medical staff during activities | 47 | Participate but do not create | 65 | Unclear conversation contents |
| 12 | Cannot share tools | 30 | Have its own manners | 48 | Pleased to be praised | 66 | Unstable work |
| 13 | Concentrate on work | 31 | Have negative remarks (e.g. "I cannot.") | 49 | Punctual | 67 | Untidy at working area |
| 14 | Create work at a high degree of perfection | 32 | High self-esteem | 50 | Refuse suggestions | 68 | Use multiple colors when creating artworks |
| 15 | Difficulty in choosing activities | 33 | Impaired body schema | 51 | Repetition of the same behavior | 69 | Work on your own without using a textbook |
| 16 | Difficulty in continuously participating in OT | 34 | Lack of completion | 52 | Restless | 70 | Work only at your own pace |
| 17 | Difficulty in finishing work | 35 | Lack of manual dexterity | 53 | Roundabout way of conversation | 71 | Work too far ahead |
| 18 | Difficulty in handling some tools | 36 | Lapses in the process (ex; OT notes incomplete) | 54 | Satisfaction with OT |  |  |

OPC, occupational performance characteristic; OT, occupational therapy

The order of listing is A to Z.
